# Substance P Hinders Bile Acid-Induced Hepatocellular Injury by Modulating Oxidative Stress and Inflammation

**DOI:** 10.3390/antiox11050920

**Published:** 2022-05-07

**Authors:** Dahyeon Lee, Jeong Seop Park, Doyoung Kim, Hyun Sook Hong

**Affiliations:** 1 Department of Biomedical Science and Technology, Graduate School, Kyung Hee University, Seoul 02447, Korea; mam102493@khu.ac.kr (D.L.); godjs@khu.ac.kr (J.S.P.); a0az515@khu.ac.kr (D.K.); 2 East-West Medical Research Institute, Kyung Hee University, Seoul 02447, Korea; 3 Kyung Hee Institute of Regenerative Medicine (KIRM), Medical Science Research Institute, Kyung Hee University Medical Center, Seoul 02447, Korea

**Keywords:** bile acid, hepatocyte, substance P, inflammation, sinusoidal endothelium

## Abstract

Liver failure is an outcome of chronic liver disease caused by steatohepatitis and cholestatic injury. This study examined substance P (SP) effect on liver injury due to cholestatic stress caused by excessive bile acid (BA) accumulation. Chenodeoxycholic acid (CDCA) was added to HepG2 cells to induce hepatic injury, and cellular alterations were observed within 8 h. After confirming BA-mediated cellular injury, SP was added, and its restorative effect was evaluated through cell viability, reactive oxygen species (ROS)/inflammatory cytokines/endothelial cell media expression, and adjacent liver sinusoidal endothelial cell (LSEC) function. CDCA treatment provoked ROS production, followed by IL-8 and ICAM-1 expression in hepatocytes within 8 h, which accelerated 24 h post-treatment. Caspase-3 signaling was activated, reducing cell viability and promoting alanine aminotransferase release. Interestingly, hepatocyte alteration by CDCA stress could affect LSEC activity by decreasing cell viability and disturbing tube-forming ability. In contrast, SP treatment reduced ROS production and blocked IL-8/ICAM-1 in CDCA-injured hepatocytes. SP treatment ameliorated the effect of CDCA on LSECs, preserving cell viability and function. Collectively, SP could protect hepatocytes and LSECs from BA-induced cellular stress, possibly by modulating oxidative stress and inflammation. These results suggest that SP can be used to treat BA-induced liver injury.

## 1. Introduction

The liver is the largest and most essential organ responsible for the removal of waste products and foreign substances from the bloodstream, control of blood sugar levels by converting glucose into glycogen, and production of essential nutrients and hormones. These roles imply that the liver contributes significantly to maintaining homeostasis and metabolic processes in the body [1].

Liver disease is caused by various factors, including viruses, drugs, and alcohol consumption. However, there are no obvious symptoms until >70% of the liver is destroyed. Although fatigue, loss of appetite, indigestion, nausea, and vomiting are typical symptoms, it is not easy to suspect the development of liver disease based on these symptoms alone. The representatives of critical liver diseases include hepatitis, alcoholic liver disease, non-alcoholic fatty liver disease, primary biliary cirrhosis (PBC), and primary sclerosing cholangitis (PSC). These diseases are accompanied by excessive fibrosis, which leads to the development of cirrhosis and liver failure. Currently, there is no way to repair liver damage that has progressed over a long period.

The liver produces bile, which is mucus that aids in the digestion of lipids. Bile acids (BAs) are the major constituents of bile and are synthesized from cholesterol in the hepatocytes. Primary BA is comprised of cholic acid and chenodeoxycholic acid (CDCA). Primary BA is secreted into the intestine, used for digestion, and then reabsorbed as secondary BA, deoxycholic acid, or ursodeoxycholic acid (UDCA).

Some pathological conditions, including genetic defects, drug toxicity, and hepatobiliary malignancies, can interrupt bile flow in the liver. In this case, BAs stagnate in the liver, leading to cholestasis and a reduction in bile flow. This exerts a detrimental effect on parenchymal/non-parenchymal cells within the liver [2]. Under cholestasis, BAs cause endoplasmic reticulum (ER) stress, mitochondrial damage, dysfunction of hepatocyte transport proteins for BA secretion, and defects in bile synthesis [3,4,5,6,7]. Constant BA stimulation promotes cell apoptosis, which results from oxidative stress with increased reactive oxygen species (ROS) generation [8]. Moreover, BA-damaged hepatocytes produce high levels of inflammatory cytokines, including interleukin (IL)-1β, IL-8, and tumor necrosis factor–α [9,10,11,12,13].

BA hydrophobicity determines its toxicity [13]. Based on its hydrophobicity, CDCA is the most potent and harmful BA. A comparative study of the effect of diverse BAs on hepatocytes demonstrated that CDCA could promote ER stress, ROS production, and inflammation in hepatocytes within several hours, eventually leading to cell apoptosis [7]. 

Liver sinusoidal endothelial cells (LSECs) are specialized capillary endothelial cells that are involved in the maintenance of metabolic and immune homeostasis, also acting as a structural barrier [14,15,16]. Notably, LSECs are involved in the hepatic immune response by regulating leukocyte recruitment and infiltration into the tissue [16]. If hepatocytes are damaged by excessive BAs, they produce ROS and inflammatory and apoptotic factors, which might induce LSEC dysfunction by negatively affecting viability and cellular characteristics. Considering the critical role of LSECs, their functional loss is likely to exacerbate the hepatic disease.

Various treatments were attempted to inhibit cholestasis progression. The farnesoid X receptor ligand is a nuclear BA receptor, and ligand binding reduces inflammation and hepatic BA production. Fibroblast growth factor-19 mimetics exert their effects by regulating BA synthesis to maintain homeostasis [17,18]. Immunosuppressive drugs were used to ameliorate hepatic inflammation [19]. UDCA competitively removes toxic hydrophobic BA molecules from cell membranes and organs, preventing damage to hepatocytes and bile ducts [17,20,21,22]. Additionally, antioxidants were used to block cholestasis by preventing BA-induced oxidative stress [23]. Despite these trials, conventional treatment did not provide a satisfactory efficacy, and novel therapy is required.

Substance P (SP) is a neuropeptide that binds to the neurokinin receptor 1, preventing cell apoptosis and stimulating cell proliferation via Erk/Akt signaling activation [24]. SP can simultaneously control inflammation by elevating M 2 macrophage/regulatory T cell portion in the circulation and lymphoid organ, leading to inhibition of acute/chronic disease progression. Additionally, SP could protect cells against oxidative stress, which can enhance cell viability [25,26,27,28]. It has previously been observed that SP can prevent alcohol-induced hepatic apoptosis and alleviate inflammation in a bile duct ligation animal model, possibly by increasing regulatory T cells and bone marrow stem cells [27,28]. Considering previously reported SP functions, it was assumed that SP could reduce BA-induced inflammation and apoptosis, rescuing liver dysfunction by participating in regression of inflammation or oxidative stress.

The objective of this study was to determine the restorative effect of SP on hepatocytes damaged by BA in vitro. To mimic the cholestasis environment in vitro, hepatocytes were exposed to CDCA, and injured hepatocytes were treated with SP. The recovery effects of SP on damaged hepatocytes were evaluated by analyzing cellular activity, inflammatory indicators, endothelial cell media (ECM) production, and ROS production in hepatocytes. In addition, the hepatocyte secretome effect on the ability to induce LSEC vascular tube structure was assessed in vitro.

## 2. Materials and Methods

### 2.1. Materials

HepG2 cells were obtained from the American Type Culture Collection (Manassas, VA, USA). Dulbecco’s modified Eagle’s medium (DMEM) and fetal bovine serum (FBS) were purchased from Gibco (Grand Island, NY, USA). LSECs and endothelial cell medium were obtained from ScienCell Research Laboratory (Carlsbad, CA, USA). Phosphate-buffered saline, penicillin/streptomycin, and trypsin-ethylenediaminetetraacetic acid solutions were purchased from Welgene (Daegu, Korea). SP, phenylmethylsulfonyl fluoride (PMSF), and CDCA were provided by Sigma Aldrich (St. Louis, MO, USA). Anti-GAPDH, anti-ICAM-1 (EP1442Y), and anti-caspase-3 (E87) antibodies (Abcam, Cambridge, MA, USA) were also used. Anti-epithelial cadherin (E-cadherin) (24E10), anti-cleaved caspase-3 (Asp175), anti-endothelial nitric oxide synthase (eNOS), and anti-phospho-eNOS (Ser1177) antibodies and 10X cell lysis buffer were obtained from Cell Signaling Technology (Danvers, MA, USA).

### 2.2. Cell Culture

HepG2 cells were cultured in DMEM containing 10% FBS and 100 U/mL penicillin/streptomycin at 37 °C and 5% CO_2_. LSECs were cultured in endothelial cell medium (ScienCell Research, Carlsbad, CA, USA). The culture medium was changed every other day. Images were obtained using a microscope (Nikon Eclipse; Tokyo, Japan). Conditioned HepG2 medium was added to LSEC culture media at a 1:1 ratio to check the effect of hepatocytes on endothelial cells in vitro.

### 2.3. Cell Viability Assay

HepG2 (2.0 × 10^4^ cells/well) were seeded in 96-well plates and incubated at 37 °C. CDCA was administered at the indicated dose. SP was added to the HepG2 cells 8 h after CDCA treatment. To examine cell viability, WST-1 solution (Roche, Indianapolis, IN, USA) was added to each well at 10% of the total volume of the medium, and the plates were incubated for 2 h. The absorbance was measured at 450 nm using a plate reader (Molecular Devices). The cellular activity under each experimental condition was expressed as a percentage relative to the activity of the control group.

### 2.4. Western Blot

HepG2 cells and LSECs were lysed with lysis buffer/2 mM PMSF solution. Lysates were collected via centrifugation at 12,000 rpm for 20 min at 4 °C. Protein concentration was determined using a BCA protein assay kit (Thermo Fisher Scientific, Rockford, IL, USA). Lysates were denatured and electrophoresed using sodium dodecyl sulfate polyacrylamide gel electrophoresis. The separated proteins were transferred onto nitrocellulose membranes. The membrane was blocked with 5% skimmed milk and incubated with primary antibodies overnight at 4 °C. After washing with TBS containing 0.1% Tween 20, the membrane was incubated with horseradish peroxidase-conjugated secondary antibodies for 1 h at room temperature. Blots were developed using EZ-Western Lumi Pico (Dogenbio, Seoul, Korea) and visualized using Amersham imager chemiluminescence (GE Healthcare, Chicago, IL, USA). Expression levels were quantified using ImageJ software.

### 2.5. SP Administration

SP treatment was carried out 8 h post-CDCA treatment at a final dose of 100 nM. Saline was used as a control for SP treatment.

### 2.6. Cytokine Measurement of Cytokines Using Enzyme-Linked Immunosorbent Assay (ELISA)

Conditioned media was prepared via centrifugation at 1500 rpm for 5 min at 4 °C. Human IL-8 and pigment epithelium-derived factor (PEDF) concentrations were determined according to the manufacturer’s instructions (IL-8, PEDF simple stem ELISA kit; Abcam, Cambridge, MA, USA). 

### 2.7. ROS Measurement

ROS generation was detected using 2′,7′-dichlorofluorescein (Cellular ROS assay kit, Abcam, Cambridge, MA, USA). ROS intensity in HepG2 cells was expressed relative to the control group.

### 2.8. Alanine Aminotransferase (ALT)/Alkaline Phosphatase (ALP) Assay

The activity of ALT from HepG2 was detected using the ALT/ALP Activity Assay Kit (Abcam, Cambridge, MA, USA).

### 2.9. Tube Formation Assay

LSECs were cultured in ECM. To evaluate the paracrine effect of hepatocytes, conditioned medium (CM) of HepG2 cells was added to LSECs at a 1:1 ratio (HepG2 CM: Endothelial cell medium = 1:1) and incubated for 24 h. Then, 10 µL of Matrigel were evenly distributed to each μ-slide well (ibidi GmbH, Gräfelfing, Germany). LSECs (6.0 × 10^3^ cells/well) were treated with Matrigel (Corning, NY, USA) on a microslide. The formation of tube-like structures by LSECs was monitored for 16 h. Images were obtained with a microscope (Nikon Eclipse). To quantify the ability to form a tubular structure, the net/ring structure was considered as a mesh. The average edge length of the mesh was regarded as a segment. The number of meshes and length of segments was measured using ImageJ software.

### 2.10. Statistical Analysis

All data are presented as means ± standard deviation. Statistical analyses were performed using GraphPad Prism (GraphPad Software, San Diego, CA, USA). Differences were considered statistically significant at *p* < 0.05 and were interpreted to denote statistical significance (* *p* < 0.05, ** *p* < 0.01, *** *p* < 0.001). Statistical analysis was performed using an unpaired two-tailed Student’s *t*-test.

## 3. Results

### 3.1. BA Reduced Hepatocyte Cellular Activity, Accompanied by Inflammation

To determine whether the initial CDCA dose was detrimental to HepG2 cells, they were exposed to different concentrations of CDCA and incubated for 8 h or 24 h. The cell morphology, viability, and inflammatory factors in HepG2 cells were analyzed.

**Figure 1:**
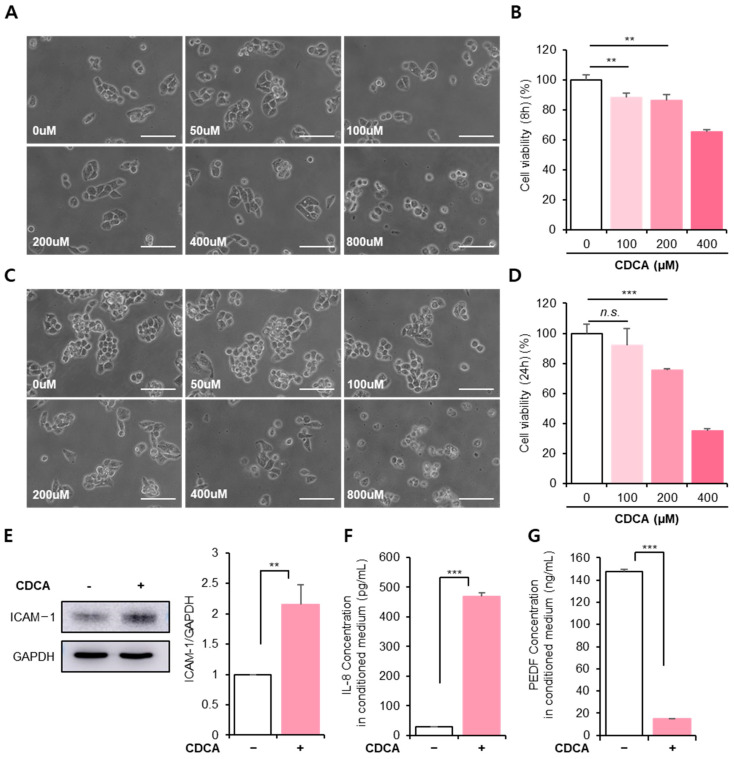
Chenodeoxycholic acid (CDCA) reduces cellular activity and induces inflammatory features in hepatocytes. CDCA effect on hepatocytes was analyzed. HepG2 cells were exposed to various concentrations of CDCA, and cell morphology and viability were evaluated 8 h (**A**,**B**) and 24 h (**C**,**D**) after CDCA treatment. Scale bar: 100 μm. Cell viability was determined using the WST-1 assay and represented relative to the control group. (**E**) ICAM-1 expression was analyzed via Western blotting at 8 h after 200 μM CDCA treatment and quantified relative to GAPDH using the Image J software. (**F**,**G**) The conditioned medium of HepG2 cells with CDCA for 8 h was collected and then analyzed for the production of interleukin (IL)-8 (**F**) and pigment epithelium-derived factor (**G**) via enzyme-linked immunosorbent assay (ELISA). The values represent the means ± standard deviations of three independent experiments. ** *p* < 0.01,*** *p* < 0.001, *n.s*: non significant.

CDCA altered cellular morphology within 8 h of treatment (Figure 1A). While 50 μM CDCA did not impair cellular shape, a dose above 100 μM seemed to moderately affect the cellular junction. Notably, 400 μM CDCA clearly disturbed cellular shape, showing a vague barrier, and 800 μM CDCA induced cell death via cell detachment. As predicted, cell viability decreased in a dose-dependent manner (Figure 1B, 100 μM: 88.25 ± 2.96%, 200 μM: 86.43 ± 4.07%, 400 μM: 65.45 ± 1.22%, 0 μM vs. 100 μM *p* < 0.01, 0 μM vs. 200 μM *p* < 0.01).

Stimulation of HepG2 cells with CDCA for 24 h exceedingly interrupted their morphology (Figure 1C). Cell viability was also reduced much more than after 8 h (Figure 1D, 100 μM: 87.03 ± 15.27%, 200 μM: 73.79 ± 0.18%, 400 μM: 35.06 ± 0.52%, 0 μM vs. 200 μM, *p* < 0.001). Consistent with cell viability, quantitative analysis for cell morphology revealed that a dose above 200 μM CDCA increased cell size with weakened cellular junction from 8 h and, 800 μM CDCA induced cell attachment, maintaining the minimal size of hepatocyte (Figure S1).

Remarkably, the 100 μM CDCA treatment was likely to cause HepG2 damage at 8 h, but this effect disappeared at 24 h. This indicates that 100 μM CDCA did not cause irreversible damage to HepG2 cells; thus, 100 μM CDCA was excluded from further experiments. As shown in Figure 1B,D, 200 μM was determined as the optimal dose of CDCA for impairing HepG2 cells.

CDCA creates an inflammatory environment in the liver by increasing the ECM expression of ICAM-1 and inflammatory cytokines [29]. These cellular changes may be the primary cause of hepatic inflammation and fibrosis. In particular, IL-8 recruits neutrophils that attach to ICAM-1 to infiltrate tissue. Thus, modulation of IL-8 and ICAM-1 may prevent the exacerbation of hepatic inflammation.

Consistent with previous reports, CDCA-induced ICAM-1 and IL-8 expression were repeatedly detected in this study (Figure 1E,F and Figure S2). Additionally, PEDF is an endogenous factor produced in hepatocytes that inhibits liver fibrosis by modulating hepatic stellate cells. In the presence of CDCA, PEDF secretion severely declined within 24 h after CDCA treatment (Figure 1G).

These results suggest that CDCA deteriorates HepG2 cellular activity and induces inflammation within 8 h. If these conditions are sustained, hepatic cell death and functional loss can occur.

### 3.2. SP Prevented CDCA-Induced Hepatic Cell Death

**Figure 2:**
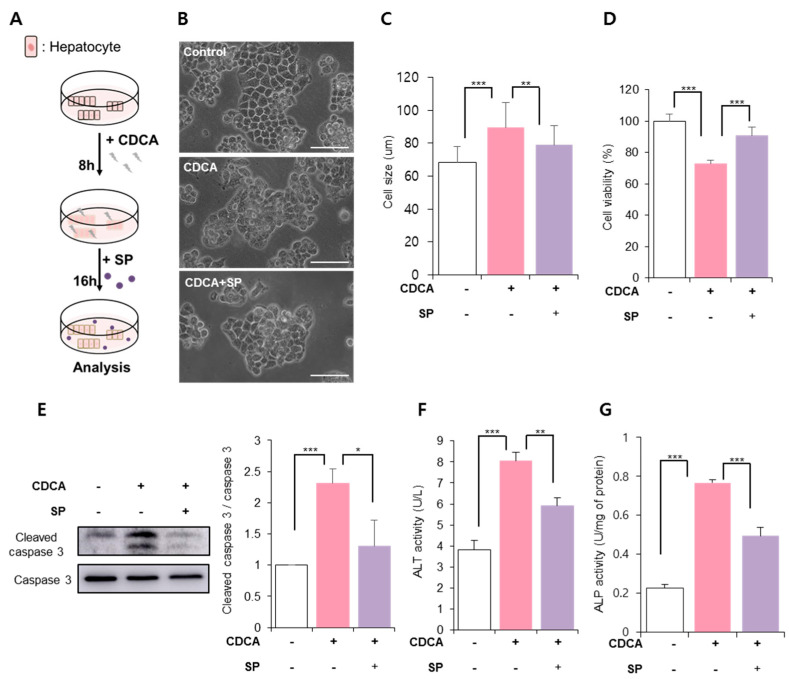
Substance P (SP) protects hepatocytes against CDCA-induced damage. The protective effects of SP against CDCA-induced hepatic injury were examined. (**A**) Experimental design for CDCA and SP treatment of HepG2 cells. HepG2 cells were treated with CDCA for 8 h, and then SP was added. After 16 h, the cell viability and cellular function of HepG2 cells were assessed. (**B**) The cellular shape of HepG2 cells treated with CDCA or SP. Scale bar: 100 μm. (**C**) Cell size was measured by image J. (**D**) The viability of HepG2 cells treated with CDCA and SP was evaluated using the WST-1 assay. (**E**) Protein levels of cleaved caspase-3 and total caspase-3 were determined via Western blotting and quantified using the Image J program. The results were compared to the total caspase-3 level. (**F**,**G**) Alanine transaminase and alkaline phosphatase activity in HepG2 cells were assayed. The values shown represent the means ± standard deviations of three independent experiments, * *p* < 0.05, ** *p* < 0.01, *** *p* < 0.001.

CDCA-mediated cellular damage was observed at 8 h after treatment and was increased at 24 h (Figure 1A). It has previously been observed that SP suppresses inflammation in the liver and restores cellular activity against diverse stressors [28,30,31]. To determine whether SP is capable of recovering hepatocytes damaged by CDCA, SP was added to HepG2 cells pretreated with CDCA for 8 h. The effect of SP was examined 24 h post-CDCA treatment (Figure 2A).

The untreated group showed a compact cellular colony with a distinct barrier, whereas CDCA treatment provoked a broken boundary and morphological alteration (Figure 2B). To some extent, CDCA-induced cellular changes were accompanied by a reduction in E-cadherin (Figure S3). However, SP-treated cells showed a more compact boundary, which led to the preservation of E-cadherin levels. Morphological analysis showed that SP treatment could alleviate enlargement of cell size due to CDCA (Figure 2C). Cell viability analysis confirmed that SP inhibited CDCA-reduced cell viability (Figure 2D, non-treated control: 100 ± 4.67%, CDCA: 72.81 ± 2.29%, CDCA + SP: 90.86 ± 5.3%; CDCA vs. CDCA + SP, *p* < 0.001).

To determine whether CDC-reduced cell viability is due to apoptosis, cleaved caspase-3 expression was assessed via Western blotting (Figure 2E). CDCA treatment elevated cleaved caspase-3 level, which was suppressed by SP treatment.

Alanine transaminase (ALT) and alkaline phosphatase (ALP) are used as representative indicators of liver damage [32]. CDCA increased ALT/ALP, but SP reduced ALT/ALP secretion in HepG2 cells treated with CDCA (Figure 2E). This corroborates the information that SP treatment can block BA-induced hepatic damage by preserving hepatic cell viability (Figure 2F,G).

### 3.3. SP Ameliorated BA-Induced Hepatic Inflammation and Oxidative Stress

**Figure 3:**
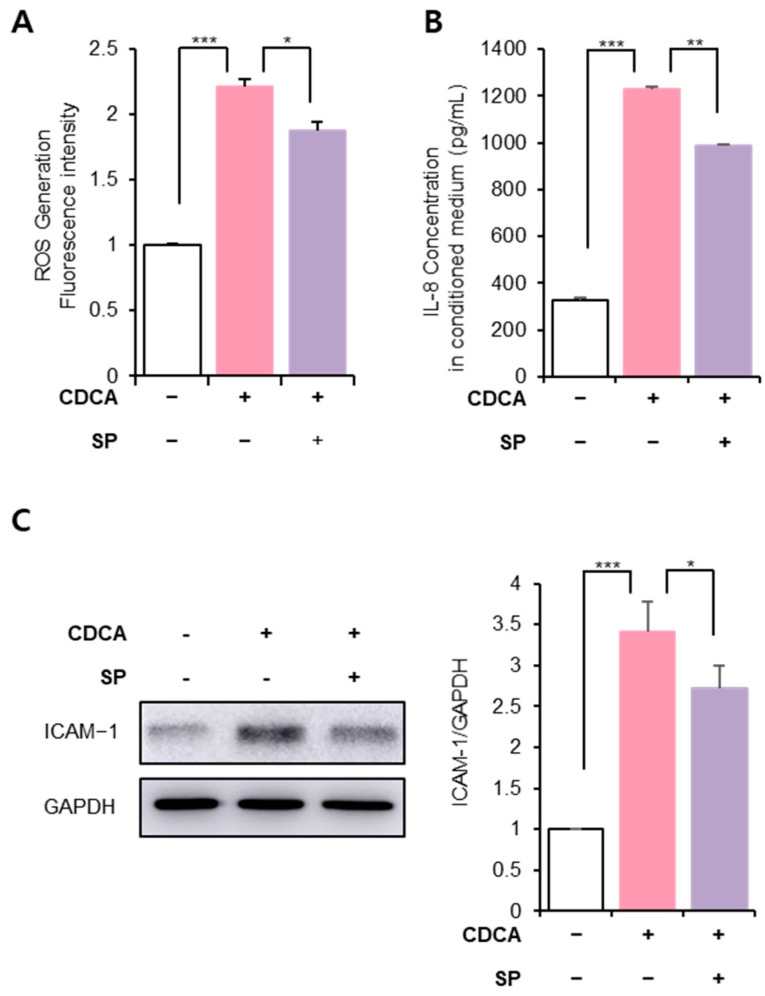
SP prevents CDCA-induced inflammation in hepatocytes. (**A**) Reactive oxygen species (ROS) generation in HepG2 cells was measured using a cellular ROS assay kit. (**B**) IL-8 concentration in HepG2 cell-conditioned media was checked using ELISA. (**C**) ICAM-1 protein level was assessed via Western blotting and quantified using the Image J program. The values shown represent the means ± standard deviations of three independent experiments, * *p* < 0.05, ** *p* < 0.01, *** *p* < 0.001.

ROS are activators of inflammatory factors in various cells, and excessive ROS production can exacerbate inflammation, eventually leading to lethal disease [29,30,31]. CDCA can enhance ROS production in hepatocytes, leading to oxidative stress, inflammation, and cellular senescence [5,33,34]. Figure 3A shows that CDCA created ROS-enriched conditions in HepG2 cells; however, SP treatment relieved this change. CDCA-induced oxidative stress is anticipated to be highly related to the induction of inflammatory factors including IL-8 and ICAM-1. At 24 h post-treatment of CDCA, IL-8 and ICAM-1 levels were extremely elevated in HepG2, which were clearly decreased in SP-treated cells (Figure 3B,C). This confirmed that the CDCA-induced pro-inflammatory condition was inhibited by SP treatment, which might occur by modulating ROS generation.

### 3.4. SP Protected the Hepatic Endothelium against BA-Damaged Hepatocyte Paracrine Action

**Figure 4:**
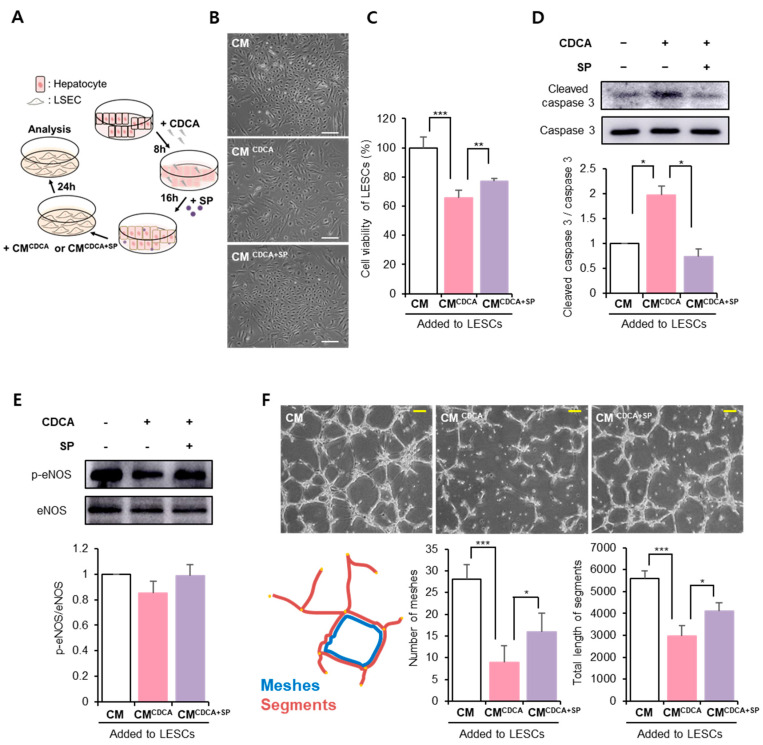
SP ameliorates LSEC injury due to secretory factors from CDCA-damaged HepG2 cells. (**A**) In order to check the effect of secretory factor of hepatocyte on function of LSECs, CM, CM^CDCA^, and CM^CDCA+SP^ were added to LSEC in a ratio of 1:1. (**B**) LSEC morphology was comparatively observed 24 h later. Scale bar: 200 μm (**C**) LSEC viability was evaluated via the WST-1 assay, and it is shown relative to the control-CM group. (**D**) Protein levels of cleaved caspase-3 in LSEC were analyzed via Western blotting and expressed relative to total caspase-3. (**E**) Protein levels of phospho-endothelial nitric oxide synthase (eNOS) were determined via Western blotting, and its expression level is shown relative to total eNOS. (**F**) LSEC ability to form a tubular structure was assessed on Matrigel, and quantification based on meshes and segments was carried out. CM: Conditioned medium of HepG2, CM^CDCA^: Conditioned medium of HepG2 with CDCA, CMCD^CA+SP^: Conditioned medium of HepG2 with CDCA and SP; Meshes: full reticulated tubes; segments: the tubes completely connected to each other. The values shown are the means ± standard deviations of three independent experiment, * *p* < 0.05, ** *p* < 0.01, *** *p* < 0.001.

LSECs are localized next to hepatocytes and maintain their structural and functional features by interacting with hepatocytes and immune cells. Thus, hepatocyte dysfunction inevitably affects LSEC activity. CDCA-treated HepG2 cells produced ROS and inflammatory cytokines (Figure 1 and Figure 3). To examine whether soluble factors from damaged hepatocytes impair LSEC viability or migratory action, the conditioned medium (CM) of HepG2 cells was used to treat LSEC for 24 h, and LSEC activity and function were evaluated (Figure 4A).

Compared to the control (HepG2 CM), CDCA-treated HepG2 CM (CM^CDCA^) expanded the cell size of LSECs with a low proliferation rate, but SP-treated CM (CM^CDCA+SP^) preserved cellular morphology, similar to the control (Figure 4B). Moreover, treatment with CM^CDCA^ reduced LSEC viability, but CM^CDCA+SP^ weakened the effect of CM^CDCA^ on LSECs (Figure 4C, Control: 100% ± 7.51%, CM^CDCA^: 68.26 ± 0.25%, CM^CDCA+SP^: 77.21 ± 1.21%, CM^CDCA^ vs. CM^CDCA+SP^ *p* < 0.01). Notably, CM^CDCA^ activated cleaved caspase-3, which might be the cause of low cellular activity, whereas CD^CDCA+SP^ showed low cleaved caspase-3 expression.

Dysfunctional endothelial cells are deficient in eNOS and NO production [35]. CM^CDCA^ decreased the levels of phospho-eNOS (p-eNOS), and CM^CDCA+SP^ tended to preserve p-eNOS levels in LSECs (Figure 4E). As a functional assay, the ability of LSECs to form tube-like structures was evaluated on Matrigel. This observation revealed that the vascular structure-forming ability was impaired by CM^CDCA^ and that CM^CDCA+SP^ enhanced this ability (Figure 4F). Quantifying tube formation revealed that the number of meshes and total length of the segments were significantly reduced by CM^CDCA^, which was restored by CM^CDCA+SP^.

This result suggests that hepatocytes with CDCA adversely affect LSECs by providing detrimental factors, but SP could ameliorate LSEC dysfunction by modulating hepatocyte activity.

## 4. Discussion

BA-induced hepatic injury was also primarily attributed to excessive ROS production. Parenchymal/non-parenchymal cells in the liver are primarily affected by ROS and reactive nitrogen species [36,37,38,39]. This process results in structural and functional liver abnormalities. Therefore, oxidative markers can be used to examine liver damage severity [36,37,38,39], and various antioxidants were used as remedies [40,41].

In this study, CDCA induced excessive generation of IL-8 and ICAM, indicating that BA-induced hepatocyte injury was associated with the initiation of inflammation. However, this alteration worsened over time. Moreover, sustained treatment with CDCA affected cell viability, promoted ALT release, and activated apoptosis signaling. In addition, PEDF production was greatly reduced.

Previous studies have shown that SP could block oxidative stress-mediated cell death in adipose-derived stem cells and retinal pigmented epithelial cells [26,30]. SP treatment of hepatocytes damaged by CDCA clearly decreased ROS production. This led to a reduction in IL-8, ICAM-1, and apoptosis levels. SP did not affect PEDF levels (Figure S4). That is, SP-mediated hepatic protection may be shown, possibly by inhibiting hepatic inflammation and cell death in the liver.

Impaired hepatocyte produces detrimental factors, causing dysfunction of neighbor endothelial cells. Treating LSEC cells with hepatocyte CM exposed with CDCA significantly impaired cellular activity and decreased the angiogenic activity of LSECs. This suggests that CDCA-mediated hepatic cell dysfunction entirely influences the vascular structure, as well as hepatocytes in the liver, leading to liver failure. In contrast, SP treatment was sufficiently able to block endothelial dysfunction, by modulating inhibition of hepatocyte alteration. Because SP was added to hepatocyte with CDCA and CM of hepatocyte was added to LESEC, it can be estimated that this CM includes SP. However, the half-life of SP is very short and thus, CM of hepatocyte with SP could not have exogenously treated SP. Therefore, the protective effect of SP on LSEC might occur, indirectly.

Collectively, SP treatment of damaged hepatocytes blocked cell death and inhibited inflammatory potential from excessive BA. SP is anticipated to be a candidate for cholestasis treatment. This study was carried out with an immortalized cell line and the confirmation of the SP effect on primary cells should be performed and the in vivo efficacy should be explored.

## Data Availability

The datasets used and/or analyzed during the present study are available from the corresponding author upon reasonable request.

## Supplementary Materials

The following supporting information can be downloaded at: https://www.mdpi.com/article/10.3390/antiox11050920/s1, Figure S1: Analysis of cellular size hepatocyte with CDCA. Figure S2: The effect of CDCA on the secretion of IL-8 on HepG2; Figure S3: SP blocks CDCA-reduced E-cadherin expression; Figure S4: The effect of SP on PEDF secretion in HepG2 under CDCA stimulation.
