# Supplementary material for: Substance P Hinders Bile Acid-Induced Hepatocellular Injury by Modulating Oxidative Stress and Inflammation
Source: Antioxidants (Basel). 2022 May 7;11(5):920. doi: 10.3390/antiox11050920 (PMC9137937; doi:10.3390/antiox11050920)
Supplement: Supplementary file 1 [file antioxidants-11-00920-s001.zip › antioxidants-1692770-supplementary.pdf]

Supplementary Figures

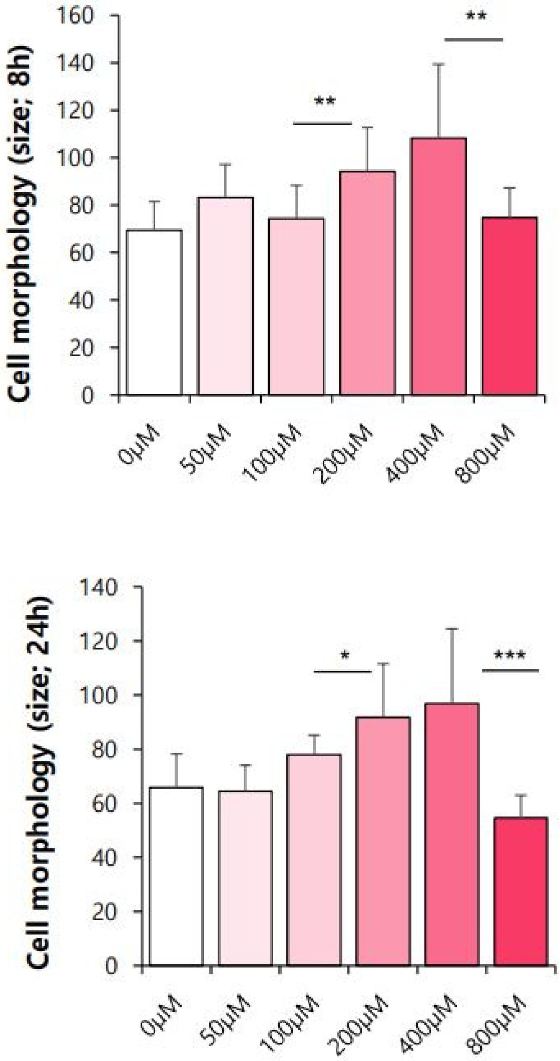

Figure S1. Analysis of cellular size hepatocyte with CDCA. HepG2 was exposed to CDCA and change of cellular morphology was analyzed by image J. The values shown are means ± standard deviations of three independent experiment, * $p < 0.05$, ** $p < 0.01$, *** $p < 0.001$.

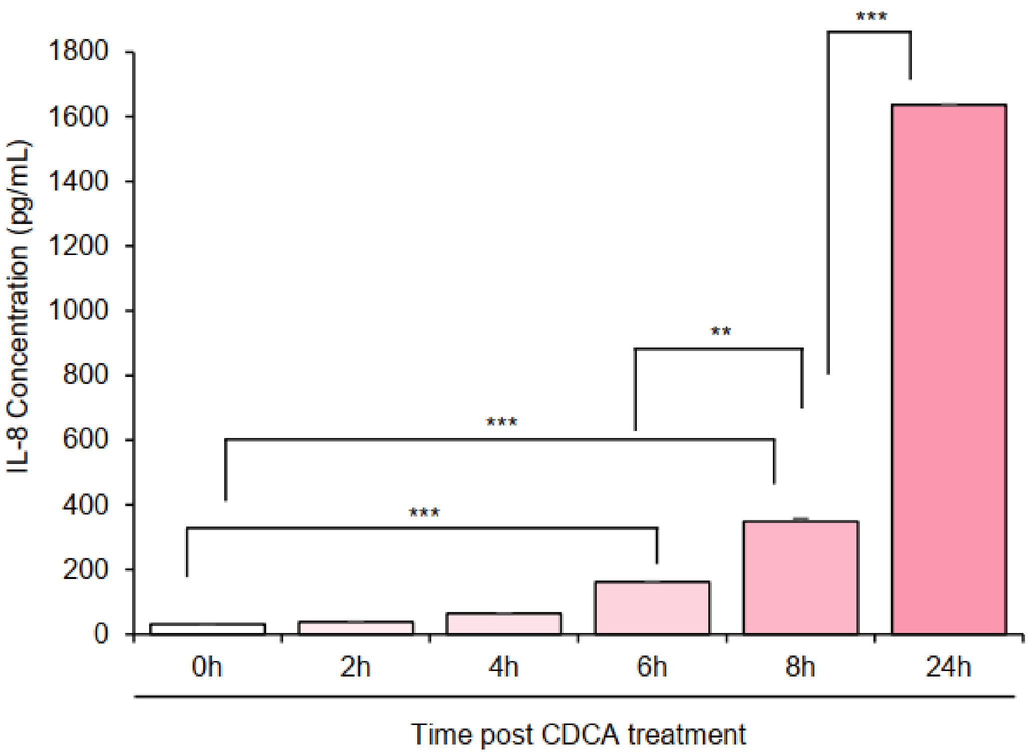

Figure S2. The effect of CDCA on secretion of IL-8 on HepG2. HepG2 was ex posed to 200μM CDCA. The CM of HepG2 was collected at 0, 2, 4, 6, 8, and 24h from CDCA treatment. Then, IL-8 concentration in CM was investigated by ELISA. The values shown are means ± standard deviations of three independent experimen t, **P < 0.01, ***P < 0.001.

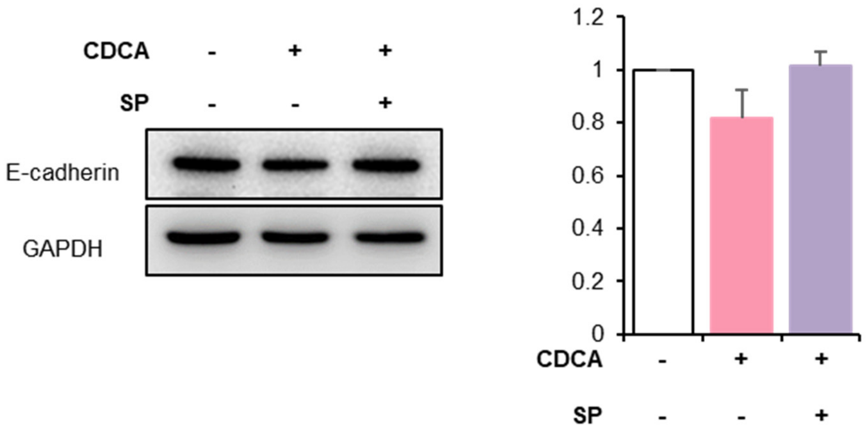

Figure S3. SP blocks CDCA-reduced E-cadherin expression. SP was treated to H epG2 that was incubated with CDCA for 8h. At 24 post CDCA treatment, HepG2 was lysed and expression of E-cadherin was analyzed by western blot. The values s hown are means ± standard deviations of three independent experiment.

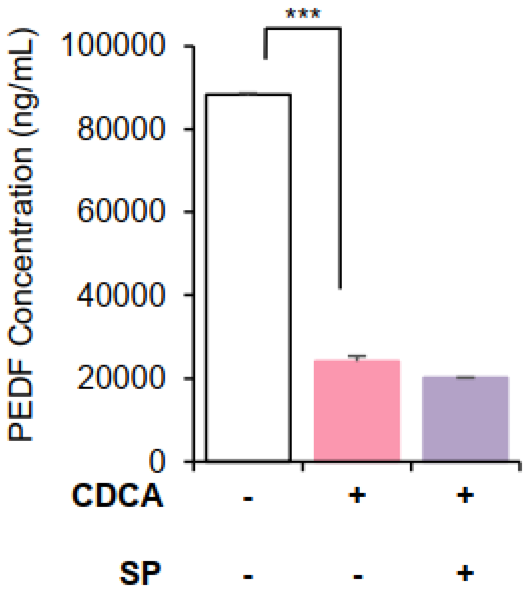

Figure S4. The effect of SP on PEDF secretion in HepG2 under CDCA stimulat ion. HepG2 was exposed to CDCA for 8h, followed by SP treatment. After 24h aft er CDCA treatment, CM of HepG2 was collected. PEDF concentration was assessed by ELISA kit. The values shown are means ± standard deviations of three independ ent experiment, ***$P < 0.001$.
